# Danggui-Yimucao Herb Pair Can Protect Mice From the Immune Imbalance Caused by Medical Abortion and Stabilize the Level of Serum Metabolites

**DOI:** 10.3389/fphar.2021.754125

**Published:** 2021-11-18

**Authors:** Shi-Jie Bi, Shi-Jun Yue, Xue Bai, Li-Mei Feng, Ding-Qiao Xu, Rui-Jia Fu, Sai Zhang, Yu-Ping Tang

**Affiliations:** Key Laboratory of Shaanxi Administration of Traditional Chinese Medicine for TCM Compatibility, and State Key Laboratory of Research and Development of Characteristic Qin Medicine Resources (Cultivation), and Shaanxi Key Laboratory of Chinese Medicine Fundamentals and New Drugs Research, and Shaanxi Collaborative Innovation Center of Chinese Medicinal Resources Industrialization, Shaanxi University of Chinese Medicine, Xi’an, China

**Keywords:** Danggui, Yimucao, medical abortion, Th1, Th2, metabonomics

## Abstract

Unintended pregnancy is a situation that every woman may encounter, and medical abortion is the first choice for women, but abortion often brings many sequelae. *Angelica sinensis* Radix (Danggui) and *Leonuri* Herba (Yimucao) are widely used in the treatment of gynecological diseases, which can regulate menstrual disorders, amenorrhea, dysmenorrhea, and promote blood circulation and remove blood stasis, but the mechanism for the treatment of abortion is not clear. We determined the ability of Danggui and Yimucao herb pair (DY) to regulate the Th1/Th2 paradigm by detecting the level of progesterone in the serum and the expression of T-bet and GATA-3 in the spleen and uterus. Then, we detected the level of metabolites in the serum and enriched multiple metabolic pathways. The arachidonic acid pathway can directly regulate the differentiation of Th1/Th2 cells. This may be one of the potential mechanisms of DY in the treatment of abortion.

## Introduction

Unintended pregnancy is a situation that all women may encounter. Since 2015, there was an average of 121 million unintended pregnancies per year, which was equivalent to a global rate of 64 unintended pregnancies per 1,000 women aged 15–49 each year. Over this period, there was an average of 73.3 million abortions per year, which corresponded to a rate of 39 abortions per 1,000 women worldwide each year. These statistics indicate that 61% of unintended pregnancies end up with abortions (Bearak et al., 2020). Abortion, as a common medical procedure, is also an important part of public health (ESHRE Capri Workshop Group, 2017; Jones and Jerman, 2017), in which medical abortion (misoprostol combined with mifepristone) is a safe, effective and highly accepted method to terminate unintended pregnancy (Jelinska and Yanow, 2018). Studies have shown that women seem to be highly receptive to medical abortions via telemedicine, and perhaps self-use of medical abortion will be promoted as a legal or recommended method (Kapp et al., 2018; Grossman, 2019). But no matter what status the medical abortion will develop, we still need to solve a series of post-abortion complications, such as endometritis and pelvic infection caused by infection, and postpartum hemorrhage (Kruse et al., 2000; Achilles et al., 2011; Costescu et al., 2016; Rouse et al., 2019). In addition, the experience of abortion may also increase the possibility of spontaneous abortion in the future, the more abortions there are, the higher the risk of spontaneous abortion (Virk et al., 2007; Nigro et al., 2011).

Danggui is the root of *Angelica sinensis* (Oliv.) Diels (Umbelliferae), which contains polysaccharides, organic acids, and phthalides (Wei et al., 2016). Its pharmacological activities are mainly immunoregulation, hematopoiesis, and antioxidant. It is often used to treat a variety of gynecological diseases that are often not easily treated with conventional therapy, such as menstrual disorders, amenorrhea, and dysmenorrhea, and is therefore known as “female ginseng” (Chen et al., 2013; Wang et al., 2016; Tian et al., 2017). Yimucao is the aerial part of *Leonurus japonicus* Houtt. (Labiatae), consisting of alkaloids, flavonoids and terpenoids (Shang et al., 2014). It has a wide range of pharmacological effects, such as protection of the uterus and heart, antioxidant and anti-tumor activities (Wang et al., 2017; Wu et al., 2018; Wang et al., 2019a), and is now mainly used in the treatment of obstetrics and gynecology diseases, including postpartum hemorrhage, postpartum persistent lochia, irregular menstruation, and subinvolution of uterus (Miao et al., 2019). The combination of the two herbs appeared as early as in a classic traditional Chinese medicine “Yimu Wan” created in ancient China (Jia et al., 2017). “Shenghua Decoction” is a classic prescription for the treatment of postpartum hemorrhage, which is recorded in the famous works “*Fu Qingzhu Nv Ke*.” On this basis, Xinshenghua granule is derived, which is also a commonly used drug in gynecology, and Pang et al. found that danggui-yimucao herb pair (DY) made outstanding contributions to Xinshenghua granule (Cheng et al., 2020a; Pang et al., 2020).

Studies had shown that drugs could regulate metabolic disorders in amino acids metabolism and lipids metabolism, and thereby alleviating the damage caused by medical abortion. Li et al. found that regulating metabolites had a certain protective effect on the uterus (Li et al., 2019; Zhang et al., 2020). As we all know, progesterone and T helper (Th) 1/Th2 were involved in maintaining pregnancy (Raghupathy, 2001), and they were also closely related to medical abortion and recurrent spontaneous abortion (Li et al., 2013a; Yuan et al., 2015). Lee et al. found that metabolites could regulate the balance of Th1/Th2 (Lee et al., 2007), Ran et al. and Li et al. also revealed that different metabolites were positively or negatively correlated with Th1 and Th2-related cytokines (Li et al., 2018; Ran et al., 2019). This study will clarify the relationship between Th1/Th2-related transcription factors/cytokines and different metabolites by establishing a connection among DY, metabonomics and Th1/Th2 paradigm, so as to explore a new direction of DY in the treatment of medical abortion. Therefore, we will detect the level of immune balance and analyze the difference of serum metabolites through UHPLC-QTOF-MS technology, so as to fully explore the potential pharmacological effects and mechanisms of DY on mifepristone (RU486)-induced abortion mice.

## Materials and Methods

### Prepare of Danggui, Yimucao, and DY

Danggui and Yimucao were purchased from Shaanxi Xingshengde Pharmaceutical Co., Ltd. (Xingshengde, Shaanxi, China) and were identified as the root of Angelica sinensis (Oliv.) Diels (No. SNTCM-20200616), and the aerial parts of Leonurus japonicus Houtt. (No. SNTCM-20200617) respectively by Prof. Yu-Ping Tang from the Shaanxi University of Chinese Medicine.

Dried Danggui (100 g), Yimucao (100 g), and DY (200 g, mass ratio was 1:1) were weighed respectively, and break them into powder. 5 times volume of 50% ethanol (v/v) was added to the powdered sample, then soaked for 20 min, ultrasonically extraction for 40 min (temperature 40°C; power 500 w), and then filtered. After extracting the residue twice with the same method, the filtrates were collected. Subsequently, the ethanol in the filtrate was recovered by vacuum distillation to obtain the Danggui, Yimucao, and DY water extract, and the water extract was concentrated for subsequent animal experiments.

### Animals

Kunming mice (male, 6–8 weeks old, bodyweight 20 ± 2 g; female, 6–8 weeks old, bodyweight 25 ± 2 g) were purchased from Chengdu Dossy Experimental Animals Co., Ltd. (Chengdu, China). All animals were kept in a temperature and light-controlled environment, 12 h light and 12 h dark cycles, and maintained with pathogen-free room with controlled conditions. All experimental procedures were approved by the Animal Care and Use Committee of Shaanxi University of Chinese Medicine. Except for the control group, all other female mice were mated with sexually mature male mice at a ratio of 2:1 overnight to establish pregnancy. The next morning, see if there was a vaginal plug. If there was, it would be designated as the 0.5 days of pregnancy.

### RU486-Induced Abortion Mouse Model and Animal Treatment

All pregnant mice were treated with RU486 (2 mg/kg; diluted with carboxymethyl cellulose; intraperitoneal injection) according to the method in the literature (Lv et al., 2012) on 8.5 days of pregnancy, pregnant mice, and the pregnancy termination rate was 100%. A cotton ball was put into the vagina for monitoring vaginal bleeding, if there was bleeding or embryo excretion, it would be considered as a successful abortion and the next step could be taken. The abortion mice were randomly divided into model group, D (Danggui group 1.04 g/ml), Y (Yimucao group 1.04 g/ml), and DY (Danggui-Yimucao group 2.08 g/ml). From the day of abortion, the mice were sacrificed after continuous administration for 7 days. The serum, spleen, and uterus were obtained respectively, and part of the fresh spleen and uterus were separated for immunohistochemistry, and the rest of samples were stored at −80°C.

### Measurement of Progesterone

The level of progesterone in mouse serum were quantified using ELISA kit according to the corresponding protocol provided by the manufacturer (Elabscience Biotechnology, Wuhan, China), and the absorbance at 450 nm was read in a microplate reader.

### Immunohistochemical Analysis

T-box expressed in T cells (T-bet) and GATA-binding protein 3 (GATA-3) expression in spleen and uterus tissues were measured by immunohistochemistry. The spleen and uterus tissues of mice were fixed in 4% paraformaldehyde, embedded in paraffin, and sectioned. The sections were processed for immunohistochemical staining with T-bet and GATA-3 monoclonal antibody (Protein Tech Group, Wuhan, China) followed by avidinbiotin based detection kit, then stained with DAB, and re-stained with hematoxylin. The images were observed under a microscope and collected for analysis.

### Quantitative Real-Time Polymerase Chain Reaction

Total RNA from spleen and uterus tissues were extracted using RNAiso plus (Takara, Japan) according to the manufacturer’s instructions, and then cDNA was generated using a reverse transcript kit (Mix) with gDNA remover (SinoMol, China). Quantitative PCR was then performed in an QuantStudioTM3 Real Time PCR System (Thermo Fisher Scientific Inc., Waltham, MA, United States) using TB Green^®^ Premix Ex Taq™ II (Tli RNaseH Plus) (Takara, Japan). All reactions were carried out in accordance with the manufacturer’s instructions. The GAPDH gene was used as the internal standard gene, and the data were quantitatively analyzed by 2^−ΔΔCT^ method. The control group was set at 1.0, and all data showed relative mRNA expression (fold change). The sequences of primers used for RT-qPCR were shown in Table 1.

**TABLE 1:** Primers sequences.

Target gene	Primer sequence (5′→3′)
T-bet	F: CTG​CCT​ACC​AGA​ACG​CAG​A
	R: AAACGGCTGGGAACAGGA
GATA-3	F: GAA​CTG​CGG​GGC​AAC​CTC​TA
	R: GCC​TTC​GCT​TGG​GCT​TGA​T
IFN-γ	F: ACA​GCA​AGG​CGA​AAA​AGG​ATG
	R: TGG​TGG​ACC​ACT​CGG​ATG​A
IL-4	F: TTG​TCA​TCC​TGC​TCT​TCT​TTC​T
	R: TGGCACATCCATCTCCGT
GAPDH	F: CCC​AGC​AAG​GAC​ACT​GAG​CAA​G
	R: GGT​CTG​GGA​TGG​AAA​TTG​TGA​GGG

### Samples Preparation

The frozen serum samples were thawed on ice, and each 100 μL of serum was mixed with 300 μL of pre-cooled acetonitrile to precipitate the protein, and then vortexed for 1 min. Each sample was centrifuged at 13,000 rpm/min for 15 min 300 μL of supernatant from each sample was taken and concentrated to dryness by vacuum centrifugation, and then redissolved with 200 μL 10% cold acetonitrile for later use. The quality control (QC) sample was mixed with 20 μL of each serum sample and processed in parallel as above. Before injection, the QC sample was injected in parallel 3 times to adjust or balance the system, and then every 10 samples were injected for further testing of the stability of the analysis system.

### UHPLC-QTOF/MS Conditions

UPLC analysis was performed on a ACQUITY UPLC system (Waters Corporation, Milford, MA, United States) that was equipped with SYNAPT G2-Si Q-TOF high-definition mass spectrometer (Waters Corp., Manchester, United Kingdom). Chromatographic separation was carried out on an UPLC BEH C18 column (2.1 × 100 mm, 1.7 µm) at 35°C. We had worked out universal UPLC elution conditions, and the conditions were as follows: mobile phase was composed of A (0.1% formic acid) and B (acetonitrile) under a gradient profile (0–1 min, 90% A; 1–2 min, 90–60% A; 2–8 min, 60–10% A; 8–12 min, 10–5% A; 12–14 min, 5–60% A; 14–15 min, 60–90% A), at a flow rate of 0.3 ml/min and a sample injection volume of 2 μL.

The UPLC system was coupled to a Q-TOF/MS using electrospray ionization (ESI) operated in both positive and negative ion modes. ESI source conditions were as follows: For positive mode, capillary voltage, 3.0 kV; source temperature, 100°C; desolvation temperature, 350°C; desolvation gas flow, 800 L/h; cone gas flow of 50 L/h. For negative mode, capillary voltage, 2.5 kV; source temperature, 120°C; desolvation temperature, 280°C; desolvation gas flow, 600 L/h; cone gas flow of 50 L/h. The MS data were automatically conducted from m/z 50 to 1,000 in the full-scan mode. Leucine encephalin at [M + H]^+^ (m/z 556.2771) and [M − H]^−^ (m/z 554.2615) was used as the lock mass.

### Data Processing and Multivariate Analysis

All of the raw UHPLC-QTOF/MS data were imported to Progenesis QI software (Waters Corporation) for peak alignment, peak selection, deconvolution and normalization. The statistical analysis was analyzed by principal component analysis (PCA) and orthogonal partial least-squares discrimination analysis (OPLS-DA) with EZinfo 3.0 software (Waters Corporation). The variable importance in the projection (VIP) > 1 in the OPLS-DA model, and *t*-test (*p* < 0.05) were used for the screening of potential biomarkers.

The m/z and retention time data provided by UHPLC-MS were analyzed by Progenesis QI software which composed of Progenesis MetaScope, HMDB (http://www.hmdb.ca/), METLIN (http://metlin.scripps.edu/) and ChemSpider (www.chemspider.com) to explore potential biomarkers. MetaboAnalyst (http://www.metaboanalyst.ca), as a web-based tool for visualization of metabolomics, was used to enrich and analyze possible metabolic pathways (Xia and Wishart, 2016).

### Statistical Analysis

Comparisons of means were conducted using one-way ANOVA, Student’s *t*-test and non-parametric test. Data were presented as the mean ± standard deviation (SD). GraphPad Prism version 8.0 (GraphPad Software, United States) was used for graphing and analyses. A value of *p* < 0.05 was considered statistically significant. Pearson correlation coefficient analysis was used to find the correlation between potential biomarkers and pharmacological indicators. Besides, RT-qPCR data were analyzed using the 2^−ΔΔCT^ algorithm.

## Results

### Effect of D, Y, and DY on the Level of Progesterone in Mice

**FIGURE 1:**
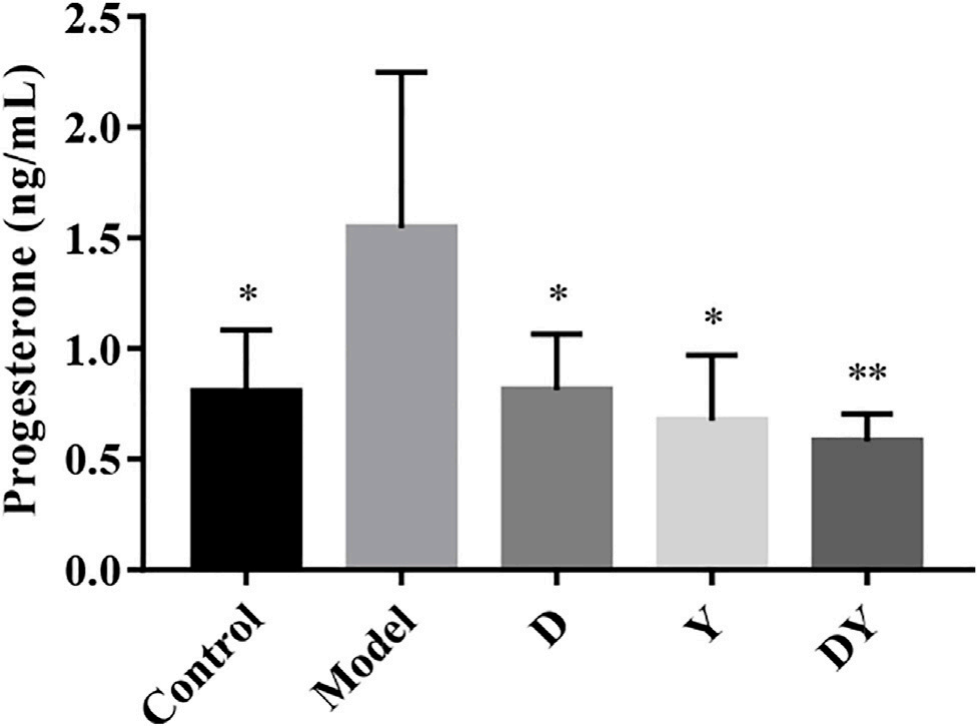
The serum progesterone levels of abortion mice treated with D, Y, and DY was measured. The data were presented as mean ± SD. (**p < 0.01 and *p < 0.05, vs. Model group).

In order to evaluate the recovery of abortion mice, we tested the serum progesterone levels of mice. As shown in Figure 1, the serum progesterone level of abortion mice was significantly higher than that of normal mice. After D, Y, and DY treatments, the progesterone levels of abortion mice were significantly reduced, and the down-regulation degree of DY group was the most obvious. The results showed that both D and Y had a certain regulatory effect on progesterone, and after the two were combined, the regulatory effect on progesterone might rise to a new level.

### Effect of D, Y, and DY on Th1/Th2 Related Transcription Factor Expression in Spleen and Uterus Tissues

**FIGURE 2:**
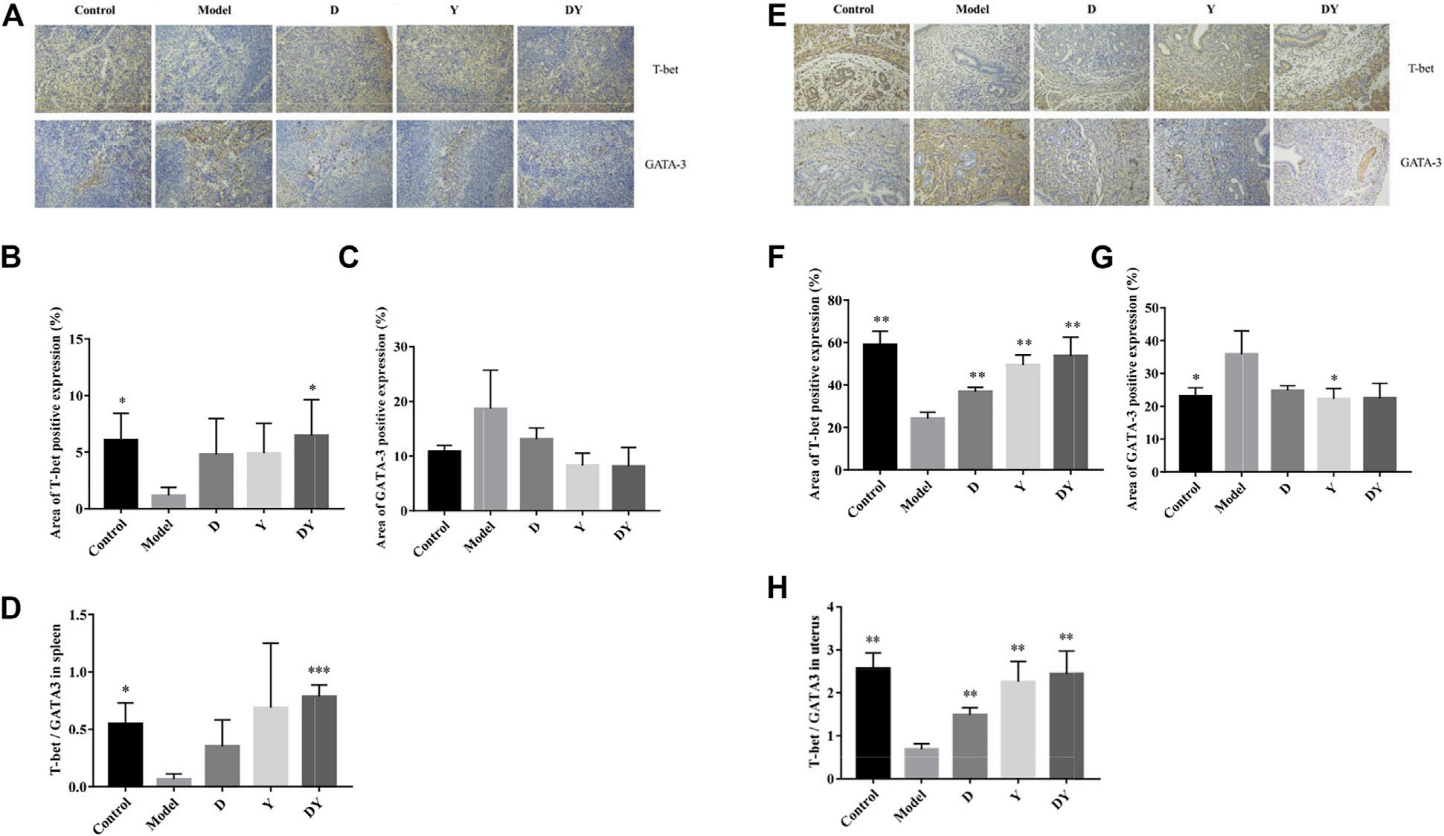
Immunohistochemical analysis of T-bet (×200) and GATA-3 (×200) expression in spleen and uterus. Staining results of spleen tissue sections **(A)**. The positive area of T-bet **(B)** and GATA-3 **(C)** in the spleen. Evaluation of T-bet/GATA-3 in the spleen **(D)**. Staining results of uterus tissue sections **(E)**. The positive area of T-bet **(F)** and GATA-3 **(G)** in the uterus. Evaluation of T-bet/GATA-3 in the uterus **(H)**. (***p < 0.001, **p < 0.01, and *p < 0.05, vs. Model group). Effect of D, Y, and DY on Th1/Th2 Related Cytokines and Transcription Factor mRNA Expression in Spleen and Uterus Tissues.

Immunohistochemical staining showed that D, Y, and DY could all regulate the expression of T-bet and GATA-3 in the spleen of abortion mice. The up-regulation of T-bet in DY group was more obvious, while the down-regulation of GATA-3 in DY and Y groups was more significant (Figures 2A–D). In addition, the DY and Y groups were better in regulating the expression of T-bet and GATA-3 in the uterus (Figures 2E–H). These results indicated that DY might have a better regulating effect on the tilt of Th1/Th2 to Th1.

**FIGURE 3:**
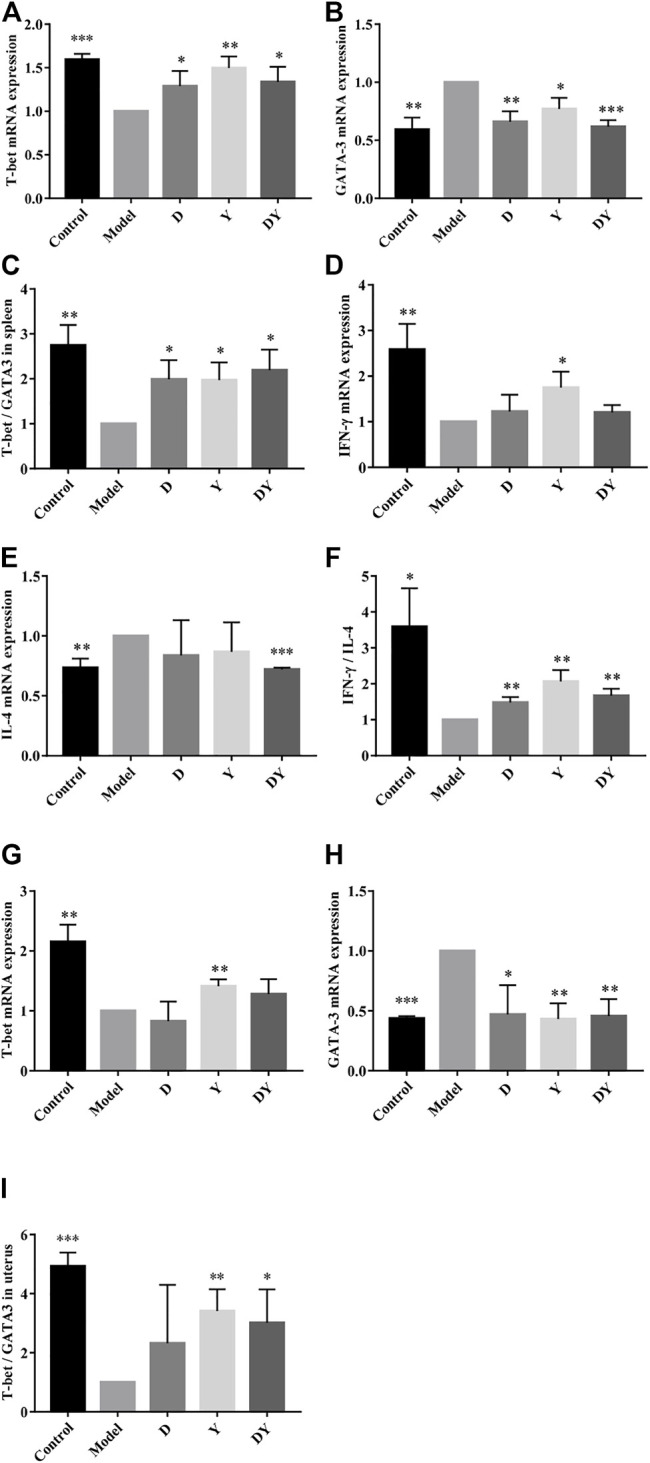
Detection of T-bet and GATA-3 mRNA expression in spleen and uterus by RT-qPCR. The mRNA expression of T-bet **(A)** and GATA-3 **(B)** in spleen. Evaluation of T-bet/GATA-3 in the spleen **(C)**. The mRNA expression of IFN-γ **(D)** and IL-4 **(E)** in spleen. Evaluation of IFN-γ/IL-4 in the spleen **(F)**. The mRNA expression of T-bet **(G)** and GATA-3 **(H)** in uterus. Evaluation of T-bet/GATA-3 in the uterus **(I)**. (***p < 0.001, **p < 0.01, and *p < 0.05, vs. Model group).

The results showed that compared with the control group, the mRNA expression of T-bet in the spleen of the model group was significantly decreased, while GATA-3 was significantly increased. After D, Y, and DY treatments, the gene expression of abortion mice had a significant correction. The Y (Figure 3A) group had a more significant up-regulation of T-bet mRNA expression, while the DY (Figure 3B) group had a better inhibitory ability on GATA-3. In addition, compared with the control group (Figures 3D,E), the expression of interferon (IFN)-γ in the model group was suppressed, while the expression of IL (interleukin)-4 was increased. After administration, the IFN-γ in Y group was significantly up-regulated, while the IL-4 in DY group was obviously inhibited. We also tested the expression of T-bet and GATA-3 in the uterus (Figures 3G,H), and the trend was similar to that in the spleen. These results showed that compared with normal mice, the immune balance of the abortion mice was tilted towards Th2, and this tilt could be reversed to Th1 after administration (Figures 3C,F).

### Metabolomics Profiling

**FIGURE 4:**
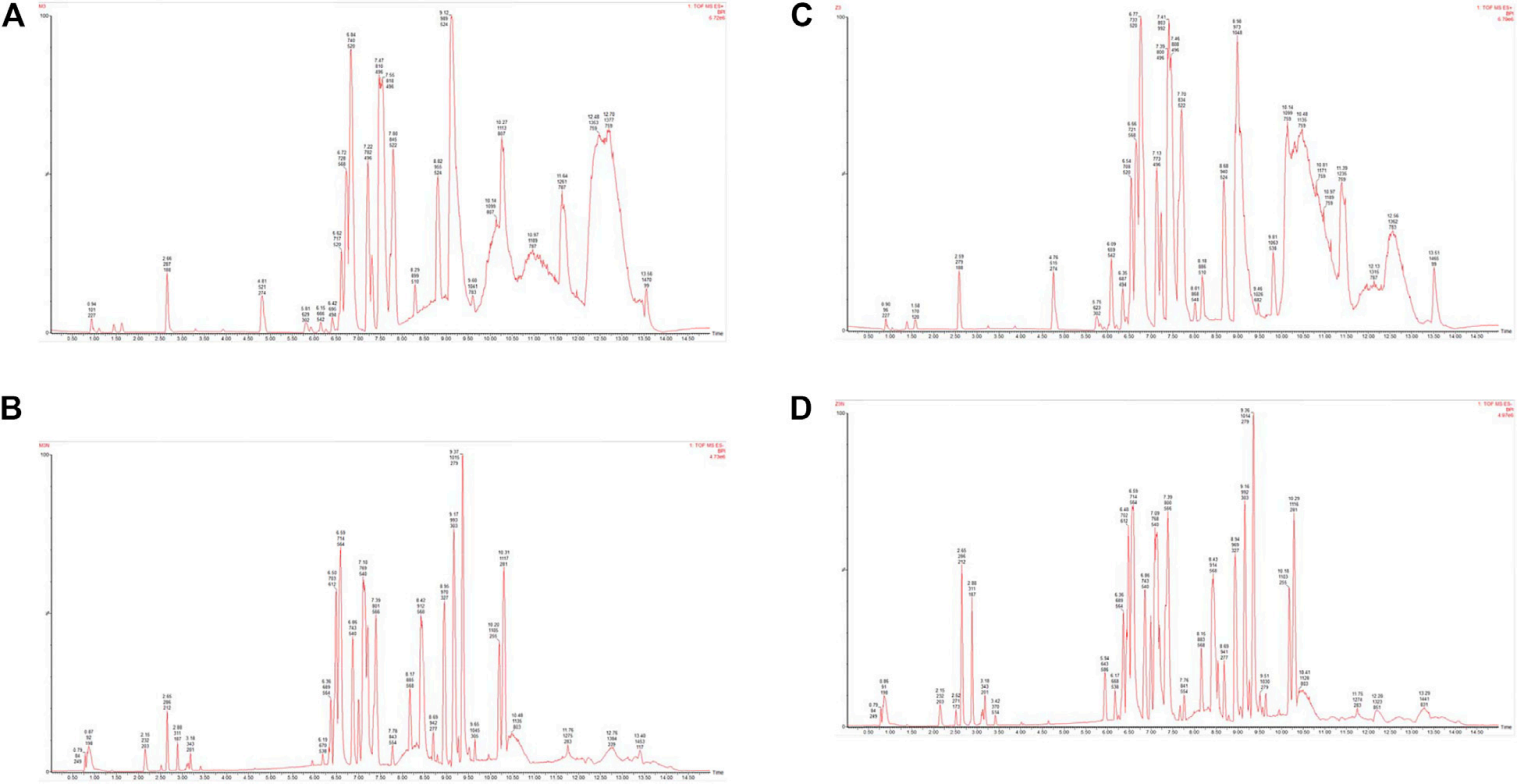
Representative BPI chromatograms of serum samples derived from the model group (**(A)**. positive; **(B)**. negative) and control group (**(C)**. positive; **(D)**. negative).

Typical base peak intensity (BPI) chromatograms of serum samples, which were collected from the model group and control group in negative and positive modes. As shown in Figure 4, the metabolites with low molecular weight could be separated well within 15 min. The subtle changes between these complex data could be found by using multivariate data analysis techniques, such as PCA and OPLS-DA.

### Multivariate Data Analysis

**FIGURE 5:**
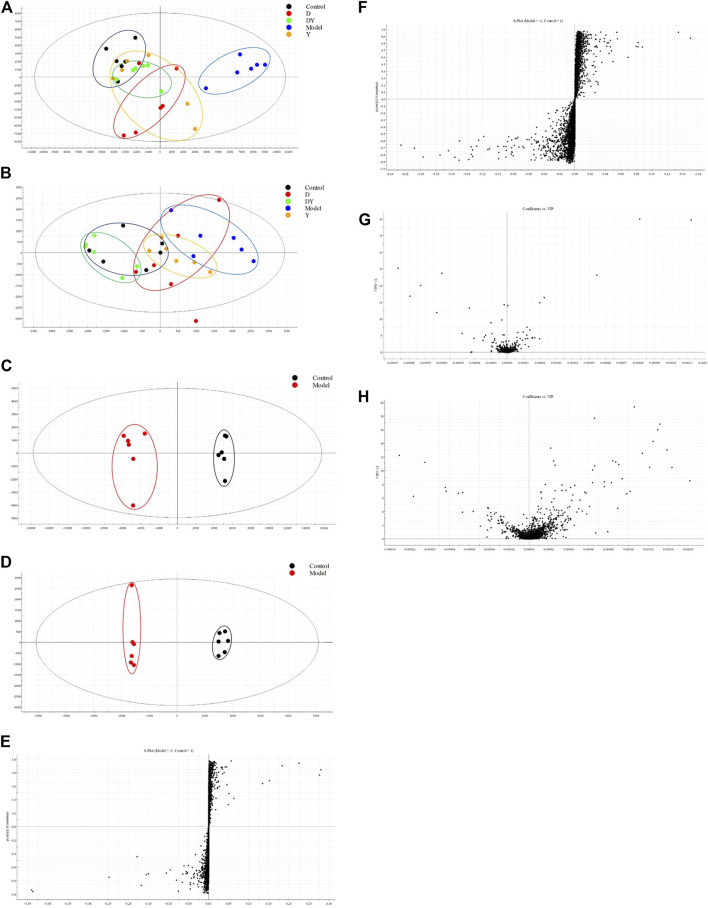
PCA score plots of serum metabolic profile in positive **(A)** and negative **(B)** ESI modes. OPLS-DA score plots of serum metabolic profile in positive **(C)** and negative **(D)** ESI modes. S-plots of serum metabolic profile in positive **(E)** and negative **(F)** ESI modes. VIP-plots of serum metabolic profile in positive **(G)** and negative **(H)** ESI modes.

According to the PCA score plot (Figures 5A,B), it could be seen that there was a clear separation between the control, model, D, Y, and DY groups. No matter in positive ion mode or negative ion mode, the model group and the control group had different trends, while D, Y, and DY groups all had different degrees of trends toward the control group, and DY group was closer to the control group.

The OPLS-DA (Figures 5C,D) was performed to further analyze the trend of the control group and the model group in positive ion mode or negative ion mode. It could be found that there were significant differences in the endogenous metabolites between the two groups from S-plots (Figures 5E,F) of OPLS-DA. Potential biomarkers could be screened by the VIP value in the VIP-value plots (Figures 5G,H), and those with a VIP value greater than 1 would be considered qualified. R^2^Y of the OPLS-DA model in positive and negative modes were 98 and 99%, and Q^2^ were 91 and 93% respectively, which showed that OPLS-DA model was good to fitness and prediction.

### Identification and Quantification of Potential Metabolites

First, potential biomarkers with high correlation were extracted from S-plots of OPLS-DA. The VIP value was often used to represent the contribution rate of variable, which was directly proportional to the VIP value. All ions detected by UPLC-MS were ranked in order of VIP value from largest to smallest, VIP > 1 and *p* < 0.05 were selected as potential biomarkers. The high-precision quasi-molecular ions detected by Q-TOF/MS and MS/MS fragmentation modes were used to calculate the possible molecular formulas of biomarkers. Progenesis MetaScope, HMDB, ChemSpider, and METLIN were performed for the verification of structural information. An endogenous (t_R_ = 9.16 min, *m/z* 303.2331) metabolite would be used as the example to illustrate the recognition process: the accurate mass of the potential marker was determined ([M-H]^−^ at *m/z* 303.2331), the main fragment ions of the marker were observed at *m/z* 285.2224, 259.2431, 231.2118, 191.1077, which might be representing [C_20_H_30_OH]^−^, [C_19_H_31_]^−^, [C_17_H_27_]^−^, and [C_12_H_17_O_2_H]^−^. The metabolite was eventually identified as arachidonic acid based on standard references and databases. In the end, a total of 76 metabolites were identified as potential biomarkers, as detailed in Supplementary Table S1 and we listed 20 of these biomarkers in Table 2. Compared with the control group, the level of serum metabolites in the model group increased or decreased to varying degrees, and the level of metabolites in abortion mice was significantly reversed after administration, especially in the DY group.

**TABLE 2:** Identification of potential markers and their changing trends among different groups (C = control, M = model).

ionization mode	No.	Description	Formula	Mass error (ppm)	Retention time (min)	*m/z*	Adducts	Trend			
								C vs. M	D vs. M	Y vs. M	DY vs. M
ESI+	1	PE (20:2 (11Z, 14Z)/18:0)	C_43_H_82_NO_8_P	1.39	9.52	794.5681	M + Na	↓**	ns	↓*	↓*
	2	Acrimarine H	C_30_H_27_NO_7_	1.24	13.35	536.1686	M + Na	↑***	ns	↑*	↑***
	3	PE (20:0/18:2 (9Z, 12Z))	C_43_H_82_NO_8_P	–4.01	12.99	794.5639	M + Na	↑**	ns	↑*	↑***
	4	LysoPC (20:4 (5Z, 8Z, 11Z, 14Z))	C_28_H_50_NO_7_P	3.54	6.75	544.3417	M + H	↓**	↓**	↓**	↓**
	5	Cer (d18:0/18:0)	C_36_H_73_NO_3_	–1.55	13.49	568.5654	M + H	↑***	ns	ns	↑**
	6	LysoPC(20:5 (5Z, 8Z, 11Z, 14Z, 17Z))	C_28_H_48_NO_7_P	2.76	6.10	542.3256	M + H, M + Na	↑**	ns	ns	↑**
	7	PE (18:4 (6Z, 9Z, 12Z, 15Z)/20:0)	C_43_H_78_NO_8_P	4.77	13.06	768.5574	M + H	↑**	ns	ns	↑***
	8	LysoPC (22:6 (4Z, 7Z, 10Z, 13Z, 16Z, 19Z))	C_30_H_50_NO_7_P	–1.17	6.68	568.3391	M + H, M + Na	↓*	↓*	↓*	↓*
	9	25-Acetyl-6,7-didehydrofevicordin F 3-glucoside	C_37_H_52_O_13_	2.82	12.26	722.3766	M + NH4	↑**	ns	ns	↑***
	10	Tetrahydro-6-(2-hydroxy-16,19-dimethylhexacosyl)-4-methyl-2H-pyran-2-one	C_34_H_66_O_3_	4.07	10.74	540.5371	M + NH4	↓***	ns	↓**	↓**
ESI-	11	Arachidonic acid	C_20_H_32_O_2_	0.60	9.16	303.2331	M − H	↓**	↓***	↓*	↓***
	12	LysoPE (0:0/22:5 (7Z, 10Z, 13Z, 16Z, 19Z))	C_27_H_46_NO_7_P	0.99	5.94	526.2944	M − H	↑***	↑**	↑***	↑***
	13	3″-Chloro-3″-deoxytriphasiol	C_19_H_23_ClO_5_	–1.03	5.94	731.2388	2M − H	↑***	↑**	↑*	↑***
	14	LysoPE (0:0/16:1 (9Z))	C_21_H_42_NO_7_P	–0.60	6.14	450.2623	M − H	↑**	↑*	↑***	↑***
	15	Formoterol	C_19_H_24_N_2_O_4_	–2.00	9.36	379.1581	M − H, M + Cl	↓**	↓**	↓*	↓***
	16	LysoPE (0:0/20:5 (5Z, 8Z, 11Z, 14Z, 17Z))	C_25_H_42_NO_7_P	0.42	5.91	498.2628	M − H	↑**	↑**	↑**	↑***
	17	2-Decylfuran	C_14_H_24_O	–2.90	5.97	253.1803	M + FA − H	↓*	↓*	↓*	↓*
	18	LysoPE (0:0/20:1 (11Z))	C_25_H_50_NO_7_P	0.70	8.56	506.3256	M − H	↑***	↑**	↑***	↑***
	19	LysoPE (0:0/18:3 (6Z, 9Z, 12Z))	C_23_H_42_NO_7_P	–0.27	5.88	474.2625	M − H	↑***	↑***	↑***	↑***
	20	Porrigenin A	C_27_H_44_O_5_	–2.48	9.36	895.6283	2M − H	↓*	↓***	↓*	↓***

***p < 0.001, **p < 0.01, and *p < 0.05.

### Metabolic Pathway Analysis

**FIGURE 6:**
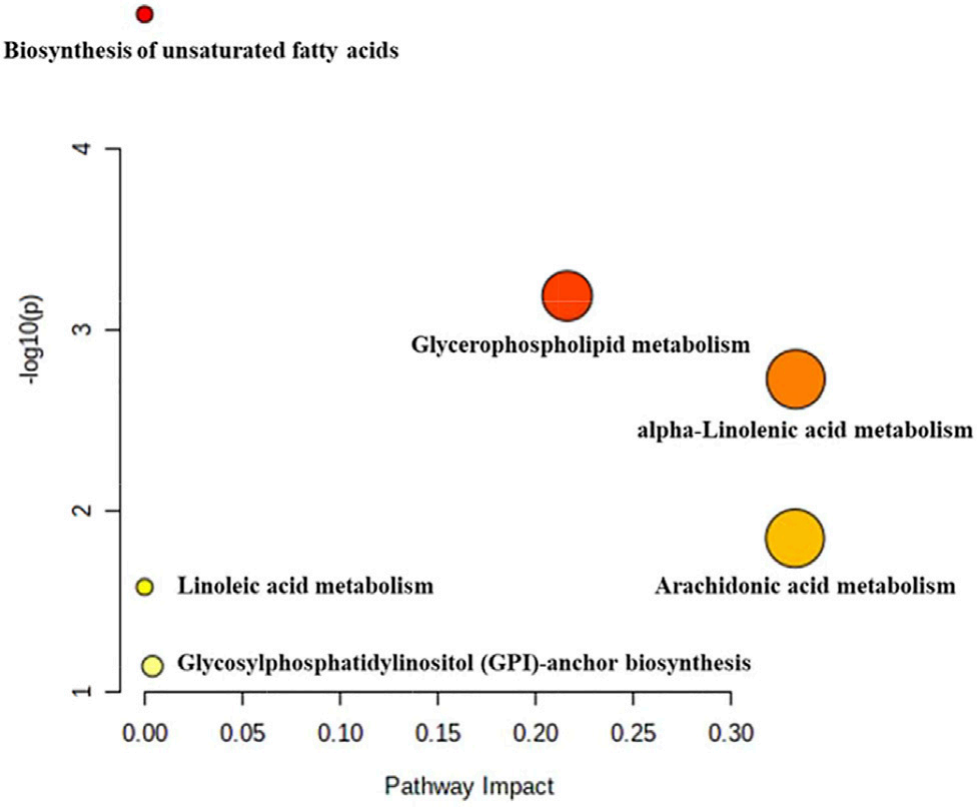
Analysis of metabolic pathways in abortion mice. The higher impact values were the glycerophospholipid metabolism, arachidonic acid metabolism, and alpha-linolenic acid metabolism respectively.

In order to analyze the effect of DY on the metabolic pathways of medical abortion mice, the metabolites identified above were introduced into MetaboAnalyst (https://www.metaboanalyst.ca/) and then pathways were constructed (Figure 6). Among them, the glycerophospholipid metabolism, Arachidonic acid metabolism, and alpha-linolenic acid metabolism, which had impact values of 0.22, 0.33, and 0.33, respectively, were selected as the most critical metabolic pathways.

As the main lipid component of cell membranes, glycerophospholipids directly affected the physiological functions of cells and were the basis for the formation of dynamic subcompartments in cell membranes, and were considered as a key molecule in cell signaling, homeostasis maintenance, inflammation and immune response (Wang et al., 2021a). They played a vital role in cell proliferation, differentiation and apoptosis. The concentration of glycerophospholipids affected the transformations in cell membrane composition and permeability. Therefore, the fluctuation of glycerophospholipids content reflected the disorder of lipid metabolism and was an important biological indicator (Wang et al., 2019b). Arachidonic acid, also known as eicosa-5, 8, 11, 14-tetraenoic acid, was a ω-6 polyunsaturated fatty acid, which mainly existed in the cell membrane in the form of phospholipids. When cells were in a state of stress, arachidonic acid was released from phospholipids as free arachidonic acids through phospholipase A_2_ and phospholipase C, and became the precursor of pro-inflammatory bioactive mediators by three metabolic pathways. Through the cyclooxygenase (COX) pathway, arachidonic acid could be metabolized into prostaglandin (PG) and thromboxane. Arachidonic acid could also be converted into leukotrienes and lipoxins through the lipoxygenase pathway. In addition, arachidonic acid also produced epoxyeicosatrienoic acid or hydroxyeicosatetraenoic acid through the cytochrome P450 pathway. These arachidonic acid metabolites were collectively called eicosanoids, which were effective autocrine and paracrine bioactive mediators and widely participated in a wide range of physiological and pathological processes (Wang et al., 2019c). There were three main metabolic pathways of alpha-linolenic acid, including ATP production and carbon cycle of β-oxidation, incorporating into glycerides within different tissues depots and converting into Long chain n-3 (Picklo and Murphy, 2016). As an arachidonic acid antagonist, increased intake of alpha-linolenic acid could lead to a decrease in arachidonic acid content and might further reduce the biosynthesis of pro-inflammatory eicosanoids, including PGE2, leukotrienes, and thromboxanes (Li et al., 2017).

### Correlation Analysis Between Biomarkers and Biochemistry Indicators

**FIGURE 7:**
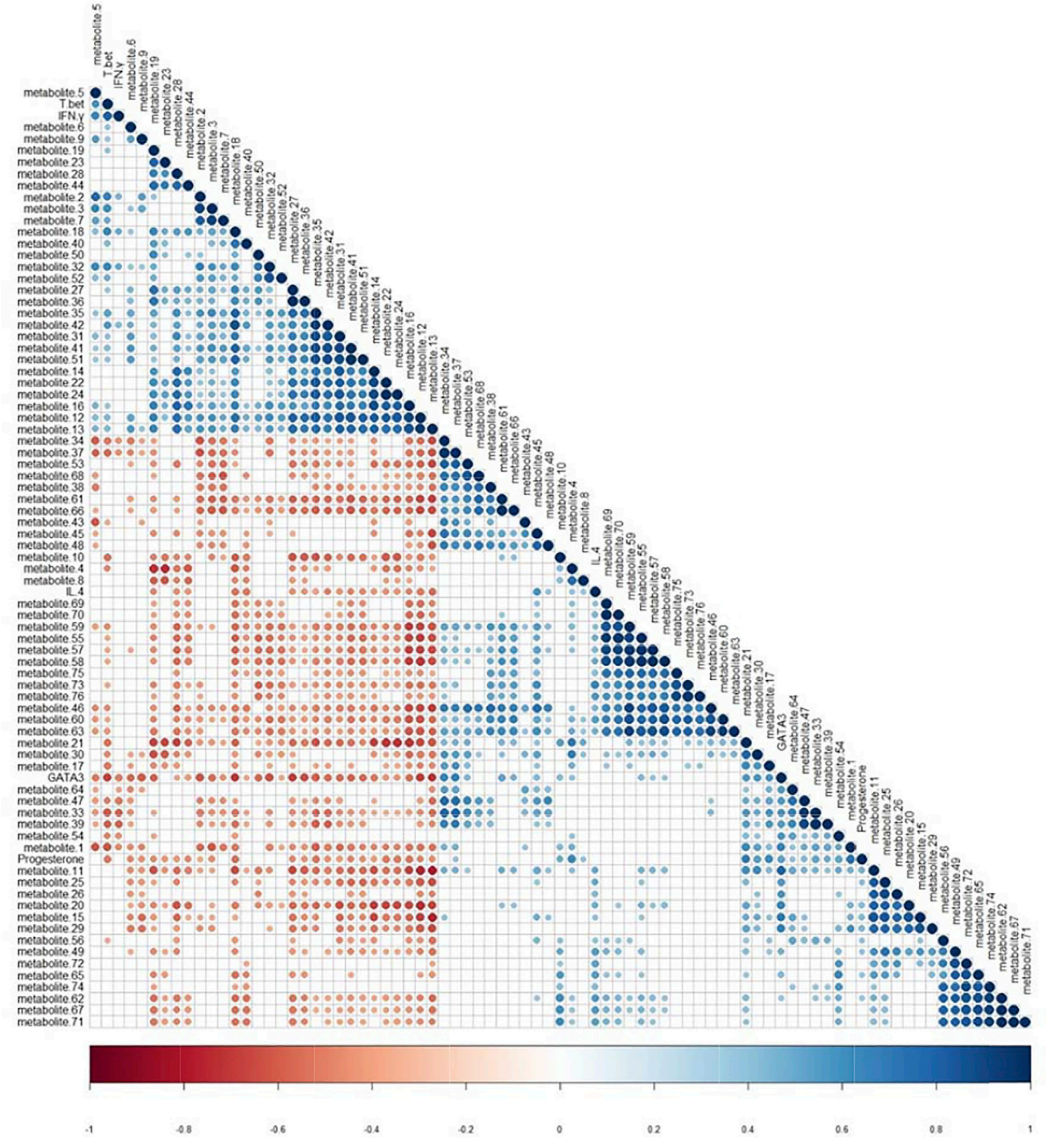
Pearson correlation coefficient analysis of biochemical indicators (progesterone, T-bet, GATA-3, IFN-γ, IL-4) and potential biomarkers (Blue: positive correlation, Red: negative correlation).

The Pearson correlation coefficient analysis method was used to find the correlation between potential biomarkers and biochemical indicators, as shown in Figure 7. Blue indicated positive correlation, while red indicated negative correlation, and the stronger the correlation, the darker the color. Progesterone had a strong positive correlation with metabolite 1 (*r* = 0.71) and GATA-3 (*r* = 0.51), and a strong negative correlation with metabolite 18 (*r* = −0.53) and T-bet (*r* = −0.51). T-bet had a strong positive correlation with metabolite 2 (*r* = 0.64), and a strong negative correlation with metabolite 1 (*r* = −0.62). GATA-3 had a strong positive correlation with metabolite 11 (*r* = 0.79), and a strong negative correlation with metabolite 13 (*r* = −0.70). The correlation coefficient was detailed in Supplementary Table S2.

## Discussion

Studies showed that Th1/Th2 was involved in both the maintenance of pregnancy and abortion (Saito et al., 2010; He et al., 2018a). The transcription factor T-bet drove Th1 differentiation, while transcription factor GATA-3 drove Th2 differentiation (Kim et al., 2014). Th1 cytokines were mainly IL-2, IFN-γ, and tumor necrosis factor (TNF)-α, while Th2 type cells mainly secreted IL-4, IL-5, and IL-10 (Azizi et al., 2019; Jiao et al., 2019). In normal pregnancy, Th2 cells and cytokines were dominant, which might be progesterone could inhibit the differentiation of Th1 but enhance the differentiation of Th2, so that Th1/Th2 balance tended to Th2 (Sykes et al., 2012a; Ahmadi et al., 2017). Li et al. found that the amount of uterine bleeding in abortion mice was closely related to progesterone and Th1/Th2 paradigm (Li et al., 2013b). Therefore, we detected the progesterone and found that compared with normal mice, the level of progesterone in the serum of abortion mice was still at a higher level, which proved that after the mice experienced an abortion, it still took some time for progesterone to fully return to normal level, and after administration, especially after DY treatment, progesterone was significantly decreased. Subsequently, the expressions of T-bet, GATA-3, IFN-γ, and IL-4 in spleen and uterus were evaluated by immunohistochemistry and qPCR, respectively. The results showed that the expression levels of Th2 transcription factors (GATA-3) and cytokines (IL-4) in abortion mice were significantly higher than those in normal mice, while Danggui and Yimucao could reverse this abnormal increase to varying degrees. In general, we thought that the regulation ability of DY was more stable and comprehensive, but single herbs also had prominent performance in some efficacy indicators.

**FIGURE 8:**
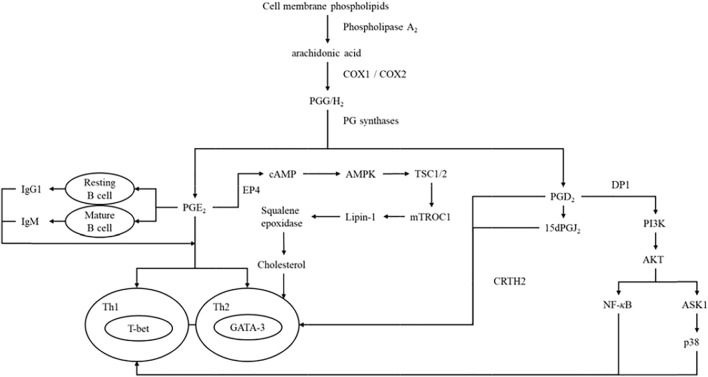
The relationship between Th1/Th2 cell differentiation and arachidonic acid metabolism pathway.

Glycerophospholipids were the key structural and regulatory components of biological membranes, as well as precursors of many active biomolecules, such as arachidonic acid and lysobisphosphatidic acid, which were catalyzed by phospholipase A_2_. PG and lysobisphosphatidic acid were the final products of glycerophospholipids, which played important roles in embryo implantation (Fu et al., 2020; Wang et al., 2021b). Arachidonic acid, a key role in abortion, was metabolized into PGF2α in the uterus and participated in various reproductive activities, such as luteolysis, maternal recognition of pregnancy, endometrial gene expression and development. The levels of arachidonic acid, PGF2α, PGE2 and thromboxane A2 in the amniotic fluid of abortion patients were significantly increased. The release of free arachidonic acid during abortion led to an increase in synthesis of PGF2α, PGE2-prostacyclin and thromboxane A2 in the fetal membranes and decidua, which might be related to abortion process of the patients (Li et al., 2021). Previous studies had shown that PGE2 inhibited the production of Th1 cytokines, but not Th2 cytokines (Betz and Fox, 1991). Arachidonic acid from cell phospholipids could be mobilized by phospholipase A_2_ (Huang et al., 2020), while PG were produced by arachidonic acid through COX and played a vital role in homeostasis and inflammation (Kabata et al., 2018), which was further metabolized by PG synthases to bioactive lipids, including PGE2 and PGD2 (Li et al., 2013c; Chandrasekharan and Sharma-Walia, 2019). PGD2 and its dehydration product 15-deoxy-Δ^12,14^-PGJ2 (15dPGJ2) acted on Th2 through chemoattractant receptor-homologous molecule expressed on Th2 cells (CRTH2) (Sykes et al., 2012b). In addition, PGD2 could bind to exogenous D-prostanoid receptor 1 (DP1), and finally acted on Th2 by regulating multiple targets. PGE2 remarkably induced the production of immunoglobulin G1 (IgG1) in resting B cells and expression of IgM in mature B cells, which in turn induced Th2 response (Wang and DuBois, 2013). PGE2 bound to PGE2 receptor 4 (EP4) to further regulate multiple targets and ultimately affected Th2 response (Kalinski, 2012; Cai et al., 2021). Details were shown in Figure 8.

DY contained many chemical components, which indicated that its action mechanism was also complex. Studies showed that *Angelica sinensis* polysaccharides promoted the proliferation of total spleen cells, macrophages, and Th cells, while the time-effect relationship of cytokine response suggested that macrophages and natural killer cells involved in non-specific immunity were activated first, and Th cells were in turn affected by polysaccharides. It could be seen that *Angelica sinensis* polysaccharides had an immunomodulatory effect by regulating the expression of Th1 and Th2 related cytokines (Chen et al., 2013; Bi et al., 2021). Volatile oil of Danggui could regulate the metabolic network with glycine and arachidonic acid as the core, and both of them were involved in the immune response (Yao et al., 2015). Chlorogenic acid, cryptochlorogenic acid, caffeic acid, and ligustilide were all potential components of Danggui for treating pelvic inflammatory disease (Zou et al., 2021). Angiogenesis was an important aspect of postpartum recovery, and the total alkaloids in Yimucao could promote it (He et al., 2018b). Leonurine could regulate the expression of PGE2 and COX2 and promote contraction of uterine smooth muscle. However, flavonoid glycosides (spinosin, linarin) in Yimucao significantly inhibited contraction of the uterine smooth muscle (Liu et al., 2018; Yin and Lei, 2018). Li et al. suggested that stachydrine could regulate the Th1/Th2/Th17/Treg paradigm by increasing the expression of T-bet and RORγt and inhibiting the expression of GATA-3 and Foxp3, ultimately reducing uterine bleeding in abortion mice. In addition, stachydrine could increase uterine contraction and promote angiogenesis, which played an important role in promoting uterine recovery (Li et al., 2013b; Cheng et al., 2020b). Zhang et al. found that senkyunolide A, ligustilide, leonurine, and ferulic acid might jointly participate in the protection of medical-induced incomplete abortion rats (Zhang et al., 2020). Our previous studies indicated that leonurine, rutin, ferulic acid, and ligustilide might be the potential components of Danggui and Yimucao regulating Th1/Th2 paradigm to relieve the immune disorders caused by medical abortion (Bi et al., 2022). Therefore, clarifying the specific components of DY in the treatment of medical abortion might also dissect the mechanism of DY, so that the target of medical abortion could be found more accurately.

This study clarified that DY could regulate the trend of Th1/Th2, thereby relieving the immune disorders caused by medical abortion. Secondly, through the enrichment analysis of serum metabolites, we obtained the key metabolic pathways for the treatment of medical abortion, such as arachidonic acid and glycerophospholipid. Subsequently, by establishing the relationship between arachidonic acid pathway and Th1/Th2 cell differentiation pathway, we speculated the potential therapeutic mechanism of medical abortion, obtained the metabolites that may play an important role, and finally provided a theoretical idea for later experimental verification. In addition, the content of alpha-linolenic acid also decreased significantly after DY treatment, indicating that the metabolism of alpha-linolenic acid was also involved in the process of medical abortion, which could also be used as a reference in subsequent studies.

## Data Availability

The original contributions presented in the study are included in the article/Supplementary Material, further inquiries can be directed to the corresponding author.
